# (*N*,*N*-Diethyl­dithio­carbamato-κ^2^ *S*,*S*′)iodido(1,10-phenanthroline-κ^2^ *N*,*N*′)copper(II)

**DOI:** 10.1107/S160053680903637X

**Published:** 2009-09-12

**Authors:** Le-Qing Fan, Ji-Huai Wu, Yun-Fang Huang, Seik Weng Ng

**Affiliations:** a Institute of Materials Physical Chemistry, Huaqiao University, Quanzhou, Fujian 362021, People’s Republic of China; b Department of Chemistry, University of Malaya, 50603 Kuala Lumpur, Malaysia

## Abstract

The copper(II) atom in the title compound, [Cu(C_5_H_10_NS_2_)I(C_12_H_8_N_2_)], is chelated by the *N*-heterocycle and the dithio­carbamate anion in a slightly distorted tetragonal coordination. The tetragonal-pyramidal coorination is completed by the iodine atom in the apical position. One ethyl group is disordered over two positions with site occupancies of 0.31 (2) and 0.69 (2).

## Related literature

For the crystal structures of other *N*,*N*′-chelated dithio­carbamatocopper adducts of *N*-heterocycles, see: Fan & Wu (2008 ▶, 2009 ▶).
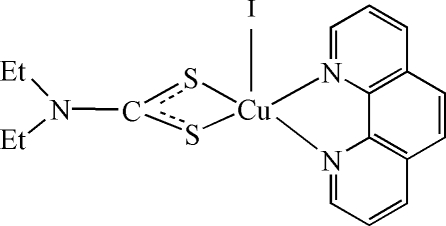


## Experimental

### 

#### Crystal data

[Cu(C_5_H_10_NS_2_)I(C_12_H_8_N_2_)]
*M* *_r_* = 518.90
Monoclinic, 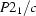

*a* = 15.357 (5) Å
*b* = 9.252 (3) Å
*c* = 14.153 (5) Å
β = 103.741 (5)°
*V* = 1953.3 (11) Å^3^
*Z* = 4
Mo *K*α radiation
μ = 2.92 mm^−1^
*T* = 293 K
0.20 × 0.20 × 0.10 mm

#### Data collection

Rigaku Mercury diffractometer
Absorption correction: multi-scan *CrystalClear* (Rigaku, 2007 ▶) *T*_min_ = 0.593, *T*_max_ = 0.759
14774 measured reflections
4470 independent reflections
3666 reflections with *I* > 2σ(*I*)
*R*_int_ = 0.033

#### Refinement

*R*[*F* ^2^ > 2σ(*F* ^2^)] = 0.043
*wR*(*F* ^2^) = 0.116
*S* = 0.93
4470 reflections
237 parameters
H-atom parameters constrained
Δρ_max_ = 0.44 e Å^−3^
Δρ_min_ = −0.73 e Å^−3^

### 

Data collection: *CrystalClear* (Rigaku, 2007 ▶); cell refinement: *CrystalClear*; data reduction: *CrystalClear*; program(s) used to solve structure: *SHELXS97* (Sheldrick, 2008 ▶); program(s) used to refine structure: *SHELXL97* (Sheldrick, 2008 ▶); molecular graphics: *X-SEED* (Barbour, 2001 ▶); software used to prepare material for publication: *publCIF* (Westrip, 2009 ▶).

## Supplementary Material

Crystal structure: contains datablocks global, I. DOI: 10.1107/S160053680903637X/bt5056sup1.cif

Structure factors: contains datablocks I. DOI: 10.1107/S160053680903637X/bt5056Isup2.hkl

Additional supplementary materials:  crystallographic information; 3D view; checkCIF report

## Figures and Tables

**Fig. 1.**
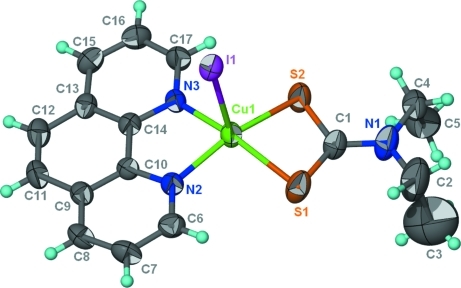
Anisotropic displacement ellipsoid plot (Barbour, 2001) of CuI(C12H8N2)(C5H10NS2) at the 50% probability level; hydrogen atoms are drawn as spheres of arbitrary radius. The minor occupied sites of the disordered ethyl chain is not shown.

## supplementary crystallographic information

### Experimental

A mixture of copper(II) acetate hydrate (0.08 g, 0.4 mmol), sodium diethyldithiocarbamate trihydrate (0.09 g, 0.4 mmol), 1,10-phenanthroline (0.08 g 0.4 mmol) and sodium iodide dihydrate (0.07 g, 0.4 mmol) was stirred in DMF (15 ml). 2-Propanol was diffused into the solution; crystals were isolated after several days, yielding single crystals.

### Refinement

Carbon-bound H-atoms were placed in calculated positions (C—H 0.93 to 0.97 Å) and were included in the refinement in the riding model approximation, with *U*(H) set to 1.2 to 1.5*U*(C).

One ethyl radical are disordered over two positions with site occupation factors of 0.31 (2):0.69 (2).

### Crystal data

**Table:**

[Cu(C_5_H_10_NS_2_)I(C_12_H_8_N_2_)]	*F*(000) = 1020
*M**_r_* = 518.90	*D*_x_ = 1.764 Mg m^−^^3^
Monoclinic, *P*2_1_/*c*	Mo *K*α radiation, λ = 0.71073 Å
Hall symbol: -P 2ybc	Cell parameters from 4020 reflections
*a* = 15.357 (5) Å	θ = 3.1–27.5°
*b* = 9.252 (3) Å	µ = 2.92 mm^−^^1^
*c* = 14.153 (5) Å	*T* = 293 K
β = 103.741 (5)°	Prism, black
*V* = 1953.3 (11) Å^3^	0.20 × 0.20 × 0.10 mm
*Z* = 4	

### Data collection

**Table:**

Rigaku Mercury diffractometer	4470 independent reflections
Radiation source: fine-focus sealed tube	3666 reflections with *I* > 2σ(*I*)
graphite	*R*_int_ = 0.033
ω scans	θ_max_ = 27.5°, θ_min_ = 2.6°
Absorption correction: multi-scan *CrystalClear* (Rigaku, 2007)	*h* = −15→19
*T*_min_ = 0.593, *T*_max_ = 0.759	*k* = −12→12
14774 measured reflections	*l* = −18→18

### Refinement

**Table:**

Refinement on *F*^2^	Primary atom site location: structure-invariant direct methods
Least-squares matrix: full	Secondary atom site location: difference Fourier map
*R*[*F*^2^ > 2σ(*F*^2^)] = 0.043	Hydrogen site location: inferred from neighbouring sites
*wR*(*F*^2^) = 0.116	H-atom parameters constrained
*S* = 0.93	*w* = 1/[σ^2^(*F*_o_^2^) + (0.0613*P*)^2^ + 3.3876*P*] where *P* = (*F*_o_^2^ + 2*F*_c_^2^)/3
4470 reflections	(Δ/σ)_max_ = 0.001
237 parameters	Δρ_max_ = 0.44 e Å^−^^3^
0 restraints	Δρ_min_ = −0.73 e Å^−^^3^

### Special details

**Table:**

Geometry. All esds (except the esd in the dihedral angle between two l.s. planes) are estimated using the full covariance matrix. The cell esds are taken into account individually in the estimation of esds in distances, angles and torsion angles; correlations between esds in cell parameters are only used when they are defined by crystal symmetry. An approximate (isotropic) treatment of cell esds is used for estimating esds involving l.s. planes.
Refinement. Refinement of F^2^ against ALL reflections. The weighted R-factor wR and goodness of fit S are based on F^2^, conventional R-factors R are based on F, with F set to zero for negative F^2^. The threshold expression of F^2^ > 2sigma(F^2^) is used only for calculating R-factors(gt) etc. and is not relevant to the choice of reflections for refinement. R-factors based on F^2^ are statistically about twice as large as those based on F, and R- factors based on ALL data will be even larger.

### Fractional atomic coordinates and isotropic or equivalent isotropic displacement parameters (Å^2^)

**Table:**

	*x*	*y*	*z*	*U*_iso_*/*U*_eq_	Occ. (<1)
Cu1	0.28574 (4)	0.83518 (6)	0.45653 (4)	0.04622 (16)	
I1	0.17818 (2)	0.58393 (3)	0.47531 (2)	0.05255 (13)	
S1	0.37150 (9)	0.78291 (19)	0.34872 (9)	0.0680 (4)	
S2	0.42493 (9)	0.78293 (18)	0.55491 (9)	0.0645 (4)	
N1	0.5328 (3)	0.6852 (6)	0.4466 (3)	0.0682 (13)	
N2	0.1835 (2)	0.9450 (4)	0.3643 (2)	0.0418 (8)	
N3	0.2373 (2)	0.9449 (4)	0.5572 (2)	0.0412 (8)	
C1	0.4550 (3)	0.7443 (5)	0.4495 (3)	0.0506 (11)	
C2	0.5523 (8)	0.6281 (16)	0.3563 (8)	0.078 (4)	0.69 (2)
H2A	0.5786	0.5327	0.3692	0.094*	0.69 (2)
H2B	0.4964	0.6180	0.3075	0.094*	0.69 (2)
C2'	0.5643 (15)	0.726 (3)	0.3480 (16)	0.065 (7)	0.31 (2)
H2D	0.5231	0.7918	0.3066	0.077*	0.31 (2)
H2E	0.6246	0.7645	0.3617	0.077*	0.31 (2)
C3'	0.559 (2)	0.569 (3)	0.305 (2)	0.113 (13)	0.31 (2)
H3D	0.6181	0.5292	0.3151	0.170*	0.31 (2)
H3E	0.5320	0.5725	0.2361	0.170*	0.31 (2)
H3F	0.5232	0.5092	0.3362	0.170*	0.31 (2)
C3	0.6108 (13)	0.716 (2)	0.3191 (10)	0.150 (8)	0.69 (2)
H3A	0.6161	0.6792	0.2574	0.224*	0.69 (2)
H3B	0.6687	0.7172	0.3636	0.224*	0.69 (2)
H3C	0.5874	0.8130	0.3109	0.224*	0.69 (2)
C4	0.6012 (3)	0.6512 (6)	0.5367 (4)	0.0620 (13)	
H4A	0.6346	0.5664	0.5257	0.074*	
H4B	0.5713	0.6284	0.5881	0.074*	
C5	0.6644 (4)	0.7723 (7)	0.5687 (5)	0.0799 (18)	
`H5B	0.6313	0.8586	0.5744	0.120*	
H5A	0.6994	0.7874	0.5217	0.120*	
H5C	0.7033	0.7495	0.6306	0.120*	
C6	0.1575 (4)	0.9422 (5)	0.2673 (3)	0.0509 (11)	
H6	0.1914	0.8907	0.2325	0.061*	
C7	0.0802 (4)	1.0150 (5)	0.2165 (3)	0.0560 (12)	
H7	0.0629	1.0093	0.1490	0.067*	
C8	0.0309 (3)	1.0935 (5)	0.2656 (4)	0.0518 (11)	
H8	−0.0206	1.1413	0.2322	0.062*	
C9	0.0586 (3)	1.1019 (4)	0.3680 (3)	0.0419 (9)	
C10	0.1346 (3)	1.0248 (4)	0.4133 (3)	0.0375 (8)	
C11	0.0123 (3)	1.1834 (5)	0.4272 (4)	0.0520 (11)	
H11	−0.0384	1.2358	0.3971	0.062*	
C12	0.0400 (3)	1.1862 (5)	0.5249 (4)	0.0512 (11)	
H12	0.0085	1.2408	0.5609	0.061*	
C13	0.1176 (3)	1.1058 (4)	0.5741 (3)	0.0425 (9)	
C14	0.1639 (3)	1.0261 (4)	0.5176 (3)	0.0383 (9)	
C15	0.1484 (4)	1.0955 (5)	0.6757 (3)	0.0521 (11)	
H15	0.1197	1.1463	0.7162	0.062*	
C16	0.2204 (4)	1.0107 (6)	0.7144 (3)	0.0558 (12)	
H16	0.2403	1.0012	0.7815	0.067*	
C17	0.2640 (3)	0.9384 (5)	0.6528 (3)	0.0489 (11)	
H17	0.3142	0.8830	0.6802	0.059*	

### Atomic displacement parameters (Å^2^)

**Table:**

	*U*^11^	*U*^22^	*U*^33^	*U*^12^	*U*^13^	*U*^23^
Cu1	0.0399 (3)	0.0589 (3)	0.0391 (3)	0.0118 (2)	0.0078 (2)	−0.0028 (2)
I1	0.0471 (2)	0.0563 (2)	0.0574 (2)	0.00439 (14)	0.01878 (15)	0.00145 (13)
S1	0.0460 (7)	0.1134 (12)	0.0443 (6)	0.0092 (7)	0.0103 (5)	−0.0187 (7)
S2	0.0442 (7)	0.1012 (11)	0.0461 (6)	0.0208 (7)	0.0069 (5)	−0.0011 (6)
N1	0.040 (2)	0.098 (4)	0.067 (3)	0.007 (2)	0.015 (2)	−0.017 (2)
N2	0.040 (2)	0.0471 (19)	0.0377 (18)	0.0040 (16)	0.0087 (15)	0.0007 (14)
N3	0.0367 (19)	0.0460 (19)	0.0403 (18)	0.0073 (15)	0.0080 (15)	0.0029 (14)
C1	0.041 (2)	0.059 (3)	0.052 (3)	−0.001 (2)	0.013 (2)	−0.013 (2)
C2	0.073 (7)	0.090 (9)	0.076 (7)	0.025 (6)	0.027 (5)	−0.016 (7)
C2'	0.055 (12)	0.071 (15)	0.079 (14)	0.012 (10)	0.038 (10)	−0.008 (11)
C3'	0.18 (3)	0.085 (19)	0.090 (19)	0.055 (19)	0.072 (19)	0.024 (14)
C3	0.161 (16)	0.207 (19)	0.099 (10)	−0.075 (14)	0.066 (10)	0.007 (10)
C4	0.044 (3)	0.057 (3)	0.086 (4)	0.004 (2)	0.017 (3)	0.002 (3)
C5	0.063 (4)	0.087 (4)	0.083 (4)	−0.018 (3)	0.006 (3)	0.008 (3)
C6	0.059 (3)	0.055 (3)	0.039 (2)	0.001 (2)	0.012 (2)	−0.0020 (19)
C7	0.064 (3)	0.057 (3)	0.040 (2)	0.002 (3)	−0.002 (2)	0.006 (2)
C8	0.051 (3)	0.043 (2)	0.056 (3)	0.004 (2)	0.001 (2)	0.010 (2)
C9	0.039 (2)	0.039 (2)	0.046 (2)	−0.0002 (18)	0.0063 (18)	0.0026 (17)
C10	0.036 (2)	0.0368 (19)	0.039 (2)	−0.0013 (17)	0.0071 (16)	0.0020 (16)
C11	0.043 (3)	0.044 (2)	0.068 (3)	0.008 (2)	0.012 (2)	0.003 (2)
C12	0.048 (3)	0.042 (2)	0.064 (3)	0.006 (2)	0.015 (2)	−0.008 (2)
C13	0.039 (2)	0.039 (2)	0.051 (2)	−0.0017 (18)	0.0134 (19)	−0.0040 (17)
C14	0.039 (2)	0.0357 (19)	0.041 (2)	−0.0028 (17)	0.0105 (17)	−0.0018 (16)
C15	0.058 (3)	0.056 (3)	0.047 (2)	−0.002 (2)	0.021 (2)	−0.011 (2)
C16	0.066 (3)	0.064 (3)	0.036 (2)	−0.001 (3)	0.011 (2)	−0.004 (2)
C17	0.052 (3)	0.058 (3)	0.035 (2)	0.008 (2)	0.0066 (19)	0.0001 (18)

### Geometric parameters (Å, °)

**Table:**

Cu1—N3	2.030 (4)	C4—C5	1.481 (8)
Cu1—N2	2.057 (3)	C4—H4A	0.9700
Cu1—S1	2.2916 (15)	C4—H4B	0.9700
Cu1—S2	2.3082 (14)	C5—`H5B	0.9600
Cu1—I1	2.9002 (10)	C5—H5A	0.9600
S1—C1	1.714 (5)	C5—H5C	0.9600
S2—C1	1.701 (5)	C6—C7	1.405 (7)
N1—C1	1.324 (6)	C6—H6	0.9300
N1—C2	1.477 (12)	C7—C8	1.354 (7)
N1—C4	1.481 (7)	C7—H7	0.9300
N1—C2'	1.62 (2)	C8—C9	1.412 (6)
N2—C6	1.335 (5)	C8—H8	0.9300
N2—C10	1.355 (5)	C9—C10	1.388 (6)
N3—C17	1.319 (5)	C9—C11	1.435 (6)
N3—C14	1.359 (5)	C10—C14	1.438 (5)
C2—C3	1.41 (2)	C11—C12	1.347 (7)
C2—H2A	0.9700	C11—H11	0.9300
C2—H2B	0.9700	C12—C13	1.438 (6)
C2'—C3'	1.57 (4)	C12—H12	0.9300
C2'—H2D	0.9700	C13—C14	1.400 (6)
C2'—H2E	0.9700	C13—C15	1.406 (6)
C3'—H3D	0.9600	C15—C16	1.360 (7)
C3'—H3E	0.9600	C15—H15	0.9300
C3'—H3F	0.9600	C16—C17	1.390 (6)
C3—H3A	0.9600	C16—H16	0.9300
C3—H3B	0.9600	C17—H17	0.9300
C3—H3C	0.9600		
			
N3—Cu1—N2	81.17 (14)	H3B—C3—H3C	109.5
N3—Cu1—S1	159.66 (12)	N1—C4—C5	112.4 (5)
N2—Cu1—S1	98.89 (11)	N1—C4—H4A	109.1
N3—Cu1—S2	97.04 (11)	C5—C4—H4A	109.1
N2—Cu1—S2	160.83 (11)	N1—C4—H4B	109.1
S1—Cu1—S2	76.18 (5)	C5—C4—H4B	109.1
N3—Cu1—I1	91.42 (11)	H4A—C4—H4B	107.9
N2—Cu1—I1	95.08 (11)	C4—C5—`H5B	109.5
S1—Cu1—I1	108.77 (5)	C4—C5—H5A	109.5
S2—Cu1—I1	104.06 (5)	`H5B—C5—H5A	109.5
C1—S1—Cu1	85.68 (16)	C4—C5—H5C	109.5
C1—S2—Cu1	85.43 (17)	`H5B—C5—H5C	109.5
C1—N1—C2	123.0 (6)	H5A—C5—H5C	109.5
C1—N1—C4	121.4 (4)	N2—C6—C7	121.8 (5)
C2—N1—C4	114.9 (6)	N2—C6—H6	119.1
C1—N1—C2'	112.7 (9)	C7—C6—H6	119.1
C2—N1—C2'	34.6 (8)	C8—C7—C6	120.2 (4)
C4—N1—C2'	119.6 (9)	C8—C7—H7	119.9
C6—N2—C10	118.0 (4)	C6—C7—H7	119.9
C6—N2—Cu1	129.8 (3)	C7—C8—C9	119.1 (4)
C10—N2—Cu1	112.1 (3)	C7—C8—H8	120.4
C17—N3—C14	118.3 (4)	C9—C8—H8	120.4
C17—N3—Cu1	128.4 (3)	C10—C9—C8	117.4 (4)
C14—N3—Cu1	113.1 (3)	C10—C9—C11	118.7 (4)
N1—C1—S2	123.3 (4)	C8—C9—C11	123.9 (4)
N1—C1—S1	124.2 (4)	N2—C10—C9	123.4 (4)
S2—C1—S1	112.4 (3)	N2—C10—C14	117.0 (4)
C3—C2—N1	113.3 (14)	C9—C10—C14	119.6 (4)
C3—C2—H2A	108.9	C12—C11—C9	122.0 (4)
N1—C2—H2A	108.9	C12—C11—H11	119.0
C3—C2—H2B	108.9	C9—C11—H11	119.0
N1—C2—H2B	108.9	C11—C12—C13	120.7 (4)
H2A—C2—H2B	107.7	C11—C12—H12	119.7
C3'—C2'—N1	97 (2)	C13—C12—H12	119.7
C3'—C2'—H2D	112.3	C14—C13—C15	117.1 (4)
N1—C2'—H2D	112.3	C14—C13—C12	118.1 (4)
C3'—C2'—H2E	112.3	C15—C13—C12	124.7 (4)
N1—C2'—H2E	112.3	N3—C14—C13	122.6 (4)
H2D—C2'—H2E	109.9	N3—C14—C10	116.5 (4)
C2'—C3'—H3D	109.5	C13—C14—C10	120.9 (4)
C2'—C3'—H3E	109.5	C16—C15—C13	119.6 (4)
H3D—C3'—H3E	109.5	C16—C15—H15	120.2
C2'—C3'—H3F	109.5	C13—C15—H15	120.2
H3D—C3'—H3F	109.5	C15—C16—C17	119.5 (4)
H3E—C3'—H3F	109.5	C15—C16—H16	120.3
C2—C3—H3A	109.5	C17—C16—H16	120.3
C2—C3—H3B	109.5	N3—C17—C16	122.9 (4)
H3A—C3—H3B	109.5	N3—C17—H17	118.6
C2—C3—H3C	109.5	C16—C17—H17	118.6
H3A—C3—H3C	109.5		
			
N3—Cu1—S1—C1	76.2 (4)	C1—N1—C4—C5	−90.7 (7)
N2—Cu1—S1—C1	164.8 (2)	C2—N1—C4—C5	98.3 (9)
S2—Cu1—S1—C1	3.66 (18)	C2'—N1—C4—C5	59.5 (13)
I1—Cu1—S1—C1	−96.74 (18)	C10—N2—C6—C7	−2.1 (7)
N3—Cu1—S2—C1	−164.2 (2)	Cu1—N2—C6—C7	174.0 (4)
N2—Cu1—S2—C1	−80.8 (4)	N2—C6—C7—C8	1.5 (8)
S1—Cu1—S2—C1	−3.69 (18)	C6—C7—C8—C9	0.5 (8)
I1—Cu1—S2—C1	102.57 (18)	C7—C8—C9—C10	−1.7 (7)
N3—Cu1—N2—C6	−179.5 (4)	C7—C8—C9—C11	179.1 (5)
S1—Cu1—N2—C6	21.1 (4)	C6—N2—C10—C9	0.8 (6)
S2—Cu1—N2—C6	94.5 (5)	Cu1—N2—C10—C9	−175.9 (3)
I1—Cu1—N2—C6	−88.8 (4)	C6—N2—C10—C14	−180.0 (4)
N3—Cu1—N2—C10	−3.2 (3)	Cu1—N2—C10—C14	3.2 (5)
S1—Cu1—N2—C10	−162.6 (3)	C8—C9—C10—N2	1.1 (6)
S2—Cu1—N2—C10	−89.2 (4)	C11—C9—C10—N2	−179.7 (4)
I1—Cu1—N2—C10	87.5 (3)	C8—C9—C10—C14	−178.1 (4)
N2—Cu1—N3—C17	177.5 (4)	C11—C9—C10—C14	1.2 (6)
S1—Cu1—N3—C17	−90.7 (5)	C10—C9—C11—C12	−0.5 (7)
S2—Cu1—N3—C17	−21.7 (4)	C8—C9—C11—C12	178.7 (5)
I1—Cu1—N3—C17	82.6 (4)	C9—C11—C12—C13	−0.5 (7)
N2—Cu1—N3—C14	2.6 (3)	C11—C12—C13—C14	0.8 (7)
S1—Cu1—N3—C14	94.4 (4)	C11—C12—C13—C15	−176.1 (5)
S2—Cu1—N3—C14	163.3 (3)	C17—N3—C14—C13	1.8 (6)
I1—Cu1—N3—C14	−92.3 (3)	Cu1—N3—C14—C13	177.3 (3)
C2—N1—C1—S2	168.7 (8)	C17—N3—C14—C10	−177.2 (4)
C4—N1—C1—S2	−1.5 (8)	Cu1—N3—C14—C10	−1.7 (5)
C2'—N1—C1—S2	−153.6 (11)	C15—C13—C14—N3	−1.9 (6)
C2—N1—C1—S1	−8.1 (10)	C12—C13—C14—N3	−179.0 (4)
C4—N1—C1—S1	−178.4 (4)	C15—C13—C14—C10	177.0 (4)
C2'—N1—C1—S1	29.5 (12)	C12—C13—C14—C10	−0.1 (6)
Cu1—S2—C1—N1	−172.0 (5)	N2—C10—C14—N3	−1.1 (5)
Cu1—S2—C1—S1	5.2 (3)	C9—C10—C14—N3	178.1 (4)
Cu1—S1—C1—N1	172.0 (5)	N2—C10—C14—C13	179.9 (4)
Cu1—S1—C1—S2	−5.2 (3)	C9—C10—C14—C13	−0.9 (6)
C1—N1—C2—C3	106.4 (13)	C14—C13—C15—C16	0.1 (7)
C4—N1—C2—C3	−82.7 (14)	C12—C13—C15—C16	177.0 (5)
C2'—N1—C2—C3	23.9 (18)	C13—C15—C16—C17	1.7 (7)
C1—N1—C2'—C3'	−114.7 (15)	C14—N3—C17—C16	0.1 (7)
C2—N1—C2'—C3'	1.0 (16)	Cu1—N3—C17—C16	−174.6 (4)
C4—N1—C2'—C3'	92.7 (17)	C15—C16—C17—N3	−1.8 (8)
